# A sex education program for teachers of preschool children: a quasi-experimental study in Iran

**DOI:** 10.1186/s12889-020-08826-y

**Published:** 2020-05-14

**Authors:** Jeno Martin, Hedyeh Riazi, Armin Firoozi, Maliheh Nasiri

**Affiliations:** 1 Department of Midwifery and Reproductive Health, Student Research Committee, School of Nursing and Midwifery, Shahid Beheshti University of Medical Sciences, Tehran, Iran; 2 Department of Midwifery and Reproductive Health, School of Nursing and Midwifery, Shahid Beheshti University of Medical Sciences, ValiAsr Ave., Cross of Niayesh Highway and ValiAsr, Tehran, 1996835119 Iran; 3 Department of Psychology, School of Psychology and Social Sciences, Islamic Azad University Roudehen branch, Tehran, Iran; 4 Department of Biostatics, School of Paramedical Sciences, Shahid Beheshti University of Medical Sciences, Tehran, Iran

**Keywords:** Sex education, Preschool child, Knowledge, Attitude, Teacher, Iran

## Abstract

**Background:**

Sex education is an important educational dimension. Together with families, teachers play a significant role in providing sex education to children. However, in most cases, they do not have enough information on this topic. The present study aimed to determine the effects of a preschool sex education program on preschool teachers’ knowledge and attitude.

**Methods:**

In this quasi-experimental study, 80 teachers working at preschools in Tehran, Iran, were randomly allocated to experimental and control groups. The educational program was provided in two 90-min sessions for the experimental group while the control group received no intervention. A self-designed knowledge and attitude questionnaire was completed by both groups before and 1 month after the intervention. This questionnaire evaluated knowledge and attitude in six domains of principles of sex education, sexual identity, stages of development and proper methods of sex education, sex-related questions, masturbation, and sexual abuse. Data were analysed in SPSS 18 using descriptive statistics as well as independent samples t-test, paired-samples t-test, chi-squared test, and ANCOVA at *p* < 0.05.

**Results:**

Mean scores of knowledge and attitude in all dimensions showed a significant increase in the experimental group following the educational intervention. However, no difference was observed in the control group. Following the educational intervention, mean scores of teachers’ knowledge and attitude in all six domains showed a significant difference with that of the control group (*p* < 0.001).

**Conclusions:**

Results revealed that the sex education program can promote the knowledge and attitude of preschool teachers in all domains.

**Trial registration:**

Iranian Registry of Clinical Trials: IRCT2016122320854N5. Registered on 9 March 2017. “Retrospectively registered”.

## Background

Sex education is a process whereby people obtain the required information and knowledge regarding sex and sexual identity and form their attitudes, beliefs, and values [1]. It is a dimension of education which is as important as other dimensions such as social, rational, and moral education.

At present, with a change in lifestyle and the more active presence of mothers in social activities, the role of other social environments, especially preschools, is as important as the role of families in transferring norms and appropriate patterns to children [2]. The majority of Canadian and American parents and teachers believe that parents and schools must cooperate in providing sex education to children [3]. Together with parents, teachers have an appropriate position for supporting children’s healthy development since they spend extensive time with them [4]. Accordingly, the role of education, especially teachers, is clear in providing sex education to children. However, teachers themselves require education in order to be able to provide appropriate information and behave appropriately with children on sex-related topics [5]. In societies like Iran in which this type of education is not provided in teacher education programs, teachers do not acquire sufficient knowledge or skills regarding child sex education [6]. Teachers’ lack of awareness of the process of child sexual development is an important cause of problems. They do not have enough knowledge to appropriately react to children’s sexual behaviours, consider some normal behaviours as abnormal, and confront, reprimand, or punish them when they show sexual behaviours [7]. If teachers do not have the right understanding of child’s normal sexual development, they may misinterpret their behaviours, thereby making children feel ashamed of their natural sexual development and be introduced as an abnormal child [8]. Therefore, it is important for teachers to know healthy and normal as well as abnormal sexual development and behaviours and realize children who are probably sexually abused [9]. It seems that the lack of a guideline on reacting to children’s sexual behaviour exposes teachers to judgments based on their own attitudes and sex education structure [10].

Preschool years are among the most significant years of children’s life [11]. In this period, children discover and explore their developmental and sexual desires and features alone or in interaction with others [9]. Sexual behaviors such as touching their own sexual organs, asking about the difference between girls and boys, and playing doctor are quite frequent among children [12]. There are different views about the necessity of sex education for children. Some believe that preschool years are not appropriate for starting sex education [13], depriving children of their innocence [14]. Studies show that 30% of children are sexually abused before the age of 6 [15]. As reported by the World Health Organization (WHO), 50% of children who experience one form of sexual abuse throughout their lives remain silent and live with this trauma [16]. Few studies have examined this issue in Iran. In a study conducted by the Social Prevention Office of the State Welfare Organization of Iran, the rate of child sexual abuse is 8 to 15% [17]. Lack of providing sex education for children may predispose them to sexual abuse [18]. Sexual abuse has negative effects on children, causing age-inappropriate sexual behaviour and behavioral problems in the victim. It can also cause considerable dysfunctions such as anxiety and depression [19].

To our Knowledge any study has yet examined preschool teachers’ attitude and knowledge, and the available studies have mostly investigated the opinions of teachers working at higher levels [1, 4, 7]. Thus, the present study aimed to determine the effects of a preschool sex education program for preschool teachers on their knowledge and attitude.

## Methods

### Study design and participants

The present quasi-experimental study recruited 80 preschool teachers at preschools in Tehran (40 in the experimental group and 40 in the control group).

Inclusion criteria were as follows: Being Iranian, minimum high school diploma, not having participated in any child sex education class, and being interested in participating in education classes. Exclusion criteria were unwillingness to continue participation and failing to participate in any session.

### Setting

First, Tehran was divided into five geographical zones of north, south, east, west, and center. Then, one municipality district was randomly selected from each zone. Of the list of preschools in each selected district, two preschools (one for the experimental group and the other for the control group) were randomly selected and, finally, 10 preschools were included in the study.

The sample size was calculated based on the results of a similar study with the power of 80% and a type I error of 0.05 [20], and it was initially estimated at 37 for each group. Then, considering the possibility of sample loss, 40 teachers were selected for the experimental group and 40 for the control group. Therefore, of each preschool, 8 teachers were recruited using the convenience sampling method.

After obtaining written informed consent, the questionnaire was completed by teachers in both groups. Afterward, the experimental group received the educational program in two 90-min sessions with a one-week interval. Education was provided in the form of lectures, question and answer, and group discussions in addition to PowerPoint presentations. Moreover, at the end of each session, a question and answer session was held on the topics covered in that session to resolve possible problems. The control group received no intervention. Four weeks after the intervention, the questionnaire was completed once more by the teachers in both groups and compared with the pretest.

### Program

The package for preschool child sex education was designed based on textbooks and articles relevant to sex education and considering the cultural context of the society. Then, it was given to some experts in the field of reproductive health and psychology and its content validity was confirmed upon making corrections.

The content of the first session included introduction, explaining the objectives and importance of sex education, preschool children’s stages of natural sex development, sexual identity, the role of teachers in the formation of sexual identity compatible with children’s sex, appropriate methods of providing sex education to children with regard to where to sleep, taking a bath, touching, familiarizing children with their sex organs, and emphasizing the privacy of these organs. The content of the second session included appropriate responses to children’s sex-related questions, masturbation in children and teacher’s correct reactions, and keeping children safe from sexual abuse. A summary of educational materials on signs of possible sexual abuse and its key recommendations are presented in Table 1.

**Table 1:** Educational materials on signs of possible sexual abuse and its key recommendations

**Educational materials**	• Difficulty walking or sitting • Withdrawal or chronic depression • Inappropriate sex play or premature understanding of sex • Feeling threatened by physical contact, closeness • Poor self-esteem, lack of confidence • Peer problems, lack of involvement with friends • Extreme weight change • Hysteria, lack of emotional control • Sudden preschool difficulties
**Educational materials**	• Encourage the child to draw pictures about persons that they like or do not like and then talk about it • Encourage the child to draw pictures about “good” and “bad” events • Encourage the child to tell teacher about the “good” and “bad” happenings • Encourage the child to talk about his/her physical problems • Remind the child that he or she is not responsible for unpleasant events • Seeking medical attention if indicated • Consult with kindergarten psychologist • Contact parents to inform them about the suspected abuse • Helping parents to contact social services or social emergency agency • Instruct the parents for separating the baby from suspicious persons
**Educational materials**	• Teachers should listen carefully to children’s sexual questions because they may be clues • Children are generally curious about their bodies and explore them • Touching their own sexual body parts is common and normal • Children are usually curious about adult bodies • Children may play games such as “Doctor” and touch one another’s sexual body parts

### Measurements

The data collection instruments were self-designed questionnaires for teachers’ knowledge and attitudes about children sex education.

The Knowledge Questionnaire comprised 31 questions on awareness in six domains of principles of sex education (4 items, α = o.77), sexual identity (3 items, α = 0.85), stages of sexual development and correct approaches to sex education (7 items, α = 0.79), children’s sexual questions (4 items, α = 0.82), masturbation (5 items, α = 0. 74), and child sexual abuse (8 items, α = 0.83). Questions could be marked “true”, “false”, or “I don’t know”. Scores ranged from 0 to 31, and higher scores showed a higher knowledge. Here are some examples for knowledge questions: *Children under the age of 6 need to have sex education, Sex education for children means learning about having sex* (for principles of sexual education domain); *Answers to children’s sexual issues should be postponed until after puberty, In response to children’s sexual questions, they should be distracted and the subject of the discussion must be changed* (for children’s sexual questions domain); *Pain when passing urine or stool can be a sign of sexual abuse of children, Most people who abuse children are relatives or friends* (for child sexual abuse domain); *Masturbation is accompanied by severe complications for children, In case of masturbation, the child’s reproductive system needs to be checked for infection or skin disease, Inappropriate encounter with children’s masturbation can exacerbate it* (for masturbation in children domain).

The Attitude Questionnaire comprised of 26 questions. The attitude of teachers was evaluated in six domains of principles of sex education (4 items, α = 0.82), sexual identity (3 items, α = 0.79), stages of sexual development and correct approaches to sex education (8 items, α = 0.84), children’s sexual questions (5 items, α = 0.87), masturbation (3 items, α = 0.75), and child sexual abuse (3 items, α = 0.78). Each question was answered with a five-point Likert scale from “completely disagree” (5) to “completely agree” (1). Items 24 and 25 were reverse-scored. Total scores ranged from 26 to 130 with a higher score demonstrating a better attitude. Here are some examples for attitude questions:

*In my opinion, sex education for children puts them at risk and abusing, In my opinion, sex education can cause early childhood sexual arousal* (for principles of sexual education domain); *In my opinion, in response to children’s sexual questions, we should say these are bad issues, and children should not talk about them, In my opinion, showing sexually themed movies to children is a good way to answer many of their sexual questions* (for children’s sexual questions domain); *In my opinion, forcing a child to kiss or be kissed by close relatives is okay, In my opinion, caressing children’s genital area is a kind of sexual abuse* (for child sexual abuse domain); *In my opinion, if a child is masturbating, it is necessary to punish him/her, In my opinion, children’s masturbation leads to sexual deviation* (for masturbation in children domain).

The validity of questionnaires was confirmed by content validity. The questionnaires was given to 10 faculty members of midwifery and reproductive health at Shahid Beheshti University of Medical Sciences and content validity ratio (CVR) and content validity index (CVI) were examined. CVR and CVI were 0.93 and 0.97, respectively, for the knowledge Questionnaire and 0.9 and 0.99, respectively, for the Attitude Questionnaire. Moreover, the internal consistency of the questionnaire was confirmed by Cronbach’s alpha of 0.79 and 0.77 for knowledge and attitude Questionnaire, respectively.

### Analysis

Data were analyzed in SPSS 18. Mean, range, frequency, and percentage were utilized to describe the data. To compare the two groups, paired-samples t-test, independent samples t-test, Mann-Whitney U test, chi-square test, and ANCOVA were employed at the significance level of < 0.05.

## Results

Mean age of teachers was 35.9 (standard deviation =7.93) years and 34.35 (standard deviation = 6.98) years in the experimental and control groups, respectively. Most teachers in both groups had a bachelor’s degree. In addition, 27.5% of teachers in the experimental group and 20% in the control group had degrees related to preschool teaching. The majority of teachers (25.6% in the experimental and 21.6% in the control group) had obtained information regarding sex education through personal studies and from the Internet. No significant difference was observed between the two groups in terms of demographic information. The details on other demographic information are provided in Table 2.

**Table 2:** Demographic characteristic in two groups

**Characteristic**	**Experimental Group (*n*** **= 40)**	**Control Group (*n* = 40)**	***P*-Value**
	n(%)	n(%)	
**Teacher’s Age**			
Less than 30	12 (30)	14 (35)	0.35^a^
30–35	8 (20)	9 (22.5)	
36–40	10 (25)	10 (25)	
More than 40	10 (25)	7 (17.5)	
**Education**			
Diploma	12 (30)	10 (25)	0.54^b^
Associate’s degree	9 (22.5)	9 (22.5)	
Bachelor’s degree	19 (47.5)	20 (50)	
Master’s degree	0	1 (2.5)	
**Related degree to preschool teaching**			
Yes	11 (27.5)	8 (20)	0.43^c^
No	29 (72.5)	32 (80)	
**Marital status**			
Single	18 (45)	12 (30)	0.36^d^
Married	21 (52.5)	27 (67.5)	
Divorced	1 (2.5)	1(2.5)	
**Work experience as a teacher**			
Less than 5 years	10 (25)	20 (50)	0.07^b^
5–10 years	14 (35)	12 (30)	
11–15 years	11 (27.5)	4 (10)	
More than 15 years	5 (12.5)	4 (10)	

^a^Independent t-test

^b^Mann-Whitney’s test

^c^Chi Square test

^d^Fisher test

Table 3 presents the means and standard deviations of teachers’ knowledge and attitude before and after the intervention in both groups. Results of the paired-samples t-test in the experimental group showed a significant difference in teachers’ knowledge scores before and after the educational intervention (*p* < 0.001). However, no significant difference was observed in the control group (*p* = 0.1). Results of the independent samples t-test indicated that mean attitude score before the intervention showed no significant difference between groups (*p* = 0.28), while this difference was significant after the intervention (*p* < 0.001).

**Table 3:** Knowledge and attitude before and after the intervention between the two groups

**Variable** (total score range)	**Experimental Group** **Mean ± SD**^**a**^	**Control Group** **Mean ± SD**^**a**^	**Independent T-Test Results**
**Knowledge** (0–31)			
Before the intervention	22 ± 3	21.15 ± 3.9	*P* = 0.28
After the intervention	29.47 ± 1.3	20.92 ± 3.9	*P* < 0.001
Paired t-test results	*P* < 0.001	*P* = 0.1	
**Attitude** (26–130)			
Before the intervention	94.1 ± 7	94.9 ± 7.52	*P* = 0.62
After the intervention	110.6 ± 5.6	94 ± 7	*P* < 0.001
Paired t-test results	*P* < 0.001	*P* = 0.12	

^a^Standard Deviation

Moreover, a significant difference was found in teachers’ mean attitude scores before and after the intervention in the experimental group (*p* < 0.001), but no such difference was observed in the control group (*p* = 0.12). Results of comparing the two groups showed that no significant difference existed in the mean scores of attitude before the intervention (*p* = 0.62), whereas the difference was significant after the intervention (*p* < 0.001).

The examination of dimensions of knowledge and attitude before the intervention revealed that the two groups did not show any significant differences in any dimensions. However, following the intervention, a significant difference was observed between the two groups in all domains of knowledge and attitude. Paired-samples t-test in the experimental group revealed a significant difference in all domains of knowledge and attitude from pretest to posttest (*p* < 0.001). The highest effect of education with regard to knowledge was observed in the domain of the stages of sexual development and correct approaches to sex education as well as sexual abuse (Table 4). As shown in Table 5, in terms of teachers’ attitude, the highest effect of education was found in the domains of stages of sexual development and correct approaches to sex education as well as masturbation.

**Table 4:** Comparison of the mean scores of the knowledge’s domains before and after the intervention in the two groups

**Domains (score range)**	**Group**	**Before intervention Mean ± SD**^**a**^	**After intervention Mean ± SD**^**a**^	**Paired t-test**	**Effect size**	**P**^**c**^
**Principles of Sexual education (0–4)**	Experimental Control	3.20 ± 0.82 3.20 ± 1.01 P^b^ = 1	3.82 ± 0.38 3.12 ± 1.11 P^b^ < 0.001	*P* < 0.001 *p* = 0.32	0.7	<0.001
**Sexual identity (0–3)**	Experimental Control	1.35 ± 0.83 1.52 ± 0.9 P^b^ = 0.37	2.77 ± 0.42 1.5 ± 0.93 P^b^ < 0.001	*P* < 0.001 *P* = 0.57	1.38	<0.001
**Sexual development stages and correct approaches for sex education (0–7)**	Experimental Control	4.87 ± 1.06 4.52 ± 1.06 P^b^ = 0.14	6.62 ± 0.54 4.47 ± 1.17 P^b^ < 0.001	*P* < 0.001 *P* = 0.48	1.94	<0.001
**Children’s sexual questions (0–4)**	Experimental Control	3.12 ± 0.82 2.92 ± 0.94 P^b^ = 0.31	3.87 ± 0.33 2.90 ± 0.92 P^b^ < 0.001	*P* < 0.001 *P* = 0.74	0.87	<0.001
**Masturbation in children (0–5)**	Experimental Control	3.55 ± 0.67 3.30 ± 0.79 P^b^ = 0.13	4.92 ± 0.26 3.30 ± 0.75 P^b^ < 0.001	*P* < 0.001 *P* = 1	1.49	<0.001
**child sexual abuse (0–8)**	Experimental Control	5.90 ± 1.29 5.67 ± 1.5 P^b^ = 0.47	7.45 ± 0.67 5.62 ± 1.5 P^b^ < 0.001	*P* < 0.001 *P* = 0.42	1.67	<0.001

^a^Standard Deviation ^b^Independent- samples t- test ^c^Derived from ANCOVA adjusted for baseline score

**Table 5:** Comparison of the mean scores of the Attitude’s domains before and after the intervention in the two groups

**Domains (score range)**	**Group**	**Before intervention** **Mean ± SD**^**a**^	**After intervention** **Mean ± SD**^**a**^	**Paired t-test**	**Effect size**	**P**^**c**^
Principles of sexual education (4–20)	Experimental Control	13.12 ± 2.8 13.75 ± 2.8 P^b^ = 0.32	16.95 ± 1.2 13.47 ± 2.3 P^b^ < 0.001	*P* < 0.001 *p* = 0.17	3.79	<0.001
Sexual identity (3–15)	Experimental Control	11.97 ± 1.3 11.37 ± 1.5 P^b^ = 0.3	12.95 ± 0.9 11.1 ± 1.4 P^b^ < 0.001	*P* < 0.001 *P* = 0.21	1.17	<0.001
Sexual development stages and correct approaches for sex education (8–40)	Experimental Control	28.67 ± 3.4 29.3 ± 2.7 P^b^ = 0.37	34.25 ± 2.4 29.3 ± 3 P^b^ < 0.001	*P* < 0.001 P = 1	5.34	<0.001
Children’s sexual questions (5–25)	Experimental Control	19.45 ± 1.9 20 ± 2.4 P^b^ = 0.23	21.15 ± 1.4 19.87 ± 2.1 P^b^ = 0.002	*P* < 0.001 P = 0.4	1.64	<0.001
Masturbation in children (3–15)	Experimental Control	9.3 ± 2.2 9 ± 1.32 P^b^ = 0.59	12.82 ± 1.1 8.67 ± 1.62 P^b^ < 0.001	*P* < 0.001 *P* = 0.03	4.05	<0.001
Child sexual abuse (3–15)	Experimental Control	11.57 ± 2.1 11.72 ± 1.8 P^b^ = 0.73	12.55 ± 1.3 11.57 ± 1.9 P^b^ = 0.001	*P* < 0.001 P = 0.4	1.06	<0.001

^a^Standard Deviation ^b^Independent- samples t- test ^c^ANCOVA

Results of analysis of covariance (ANCOVA) showed no relationship between demographic variables and teachers’ knowledge score (Table 6).

**Table 6:** The results of analysis of covariance (ANCOVA): the effect of education and demographic variables on teachers’ knowledge

**Variable**	**Estimated coefficients**	**Std. Error**	**T**	***p*-value**
Experimental Group	8.17	0.34	23.55	< 0.001
Teacher’s Education	0.225	0.218	1.03	0.304
Marital status	0.426	0.367	1.16	0.249
Work experience	0.058	0.032	−1.821	0.073
Number of children	−0.51	0.62	−0.83	0.41
Mean score of knowledge (pre-test)	0.715	0.051	14.04	< 0.001

According to Table 7, the examination of the effect of demographic variables on teachers’ attitude revealed that the level of education and marital status of teachers affect their attitude scores. By increasing the level of education by 1, attitude score was increased by 1.57 and the mean score of attitude of married teachers was higher than that of single teachers by 2.9.

**Table 7:** The results of analysis of covariance (ANCOVA): the effect of education and demographic variables on teacher’s attitude

**Variable**	**Estimated coefficients**	**Std. Error**	**T**	***p*-value**
Experimental Group	17.9	0.95	18.77	< 0.001
Teacher’s Education	1.57	0.57	2.71	0.008^*^
Marital status	2.90	1.013	2.86	0.005^*^
Work experience	−0.044	0.087	−0.51	0.611
Number of children	−1.16	1.45	−0.8	0.42
Mean score of Attitude (pre-test)	0.613	0.066	9.34	< 0.001

^*^P-value < 0.05

## Discussion

The present study aimed to determine the effects of preschool sex education on teachers’ knowledge and attitude in Iran. Overall the findings showed that sexual education to preschool teachers improved their knowledge and attitudes toward sex education for preschool children. However, the improvement was more evident for knowledge compared to attitude. This might indicate that changing attitude may need more time and perhaps more profound interventions.

In line with previous studies, results demonstrated that the educational intervention increased teachers’ knowledge. These studies emphasize the necessity of providing educational programs, seminars, and courses to upgrade knowledge on child sex education [21]. Other studies on education for teachers at higher levels similarly show that educational interventions enhance the level of knowledge in teachers [1, 22].

Teachers can be a reliable source for providing information on sex education to children. However, it seems that they do not possess enough awareness of sex-related matters [23]. According to Martinez et al., only 11.9% of teachers have received sex education in their postgraduate programs. In teachers’ view, the major perceived barriers to sex education are lack of proper education and resources [24]. Lack of comprehensive knowledge and awareness on child sex-related matters has caused an obvious gap in the identification of normal and problematic behaviors in children by teachers. These behaviors may not be socially acceptable in educational settings, but they are part of the normal sexual development and must be regarded as normal [8]. Teachers have inadequate knowledge of child development and cannot distinguish between their normal sexual behaviors and curiosities. Lack of awareness leads to reduced self-confidence for making judgments [25]. Teachers require education in order to provide appropriate information and behave appropriately with children on sex-related topics. Based on studies on teachers, inadequate knowledge and feeling embarrassed while discussing sex-related matters have been introduced as barriers to sex education [26, 27].

In the present study, the domains of stages of sexual development and correct approaches to sex education in teachers’ knowledge showed the highest effect, indicating that education was the most effective on this domain. As those in close contact with children, teachers need to be educated on the stages of child development - especially in relation to sexual development - and have an accurate understanding of natural stages of development in children [28]. They must be able to identify normal and abnormal sexual behaviors and respond to children based on their age [29].

The second highest effect of education was found in the domain of child sexual abuse. A study in the USA reported that, after participation in a short-term educational course, posttest knowledge scores of teacher-training students regarding child sexual abuse were significantly increased compared to pretest [30]. McKee and Dillenburger (2012) concluded that educational workshops promote teachers’ knowledge of child sexual abuse [26]. Children who have been sexually abused suffer from behavioral problems, mental obsession with sexual matters, academic underachievement, depression, anger, and guilt. Therefore, teachers who can timely identify sexual abuse in children and correctly report these behaviors play a major role in supporting them [31].

Results of the present study suggest the positive effect of sexual intervention on teachers’ attitude regarding preschool children’s sex education, which is consistent with some other studies [32]. According to Gursimsek, holding sex education courses for teacher-training students can positively affect their attitude regarding sex education. Furthermore, a positive attitude can effectively promote students’ awareness [33]. A study by Fentahun et al. revealed that 98% of participants have a positive attitude toward sex education. They believed that the educational content must be appropriate to children’s age and needs and compatible with their mental maturity [34]. Results of the study by Kenny indicate that teachers lack the required knowledge and attitude in the domains of identifying misbehaviors and neglect as well as the process of reporting these matters. They require standard education and clear and simple guidelines [35].

In the present study, education was shown to have the most effect on two domains of teachers’ attitudes, sexual development and correct approaches to sex education, and masturbation, respectively. Pasikowska evaluated teachers’ attitude and reported a negative attitude regarding preschool children’s sexual behavior. According to participants, the emergence of sexual behaviors in children indicates deviations and cause dysfunction in their development [36]. If teachers act inappropriately, confuse children, and fail to answer their questions, children turn to their peers. Mkumbo (2012) examined teachers’ attitudes regarding sex education and reported that it is not enough to show a positive attitude toward sex education, and teachers’ knowledge and awareness of various topics in sex education should also be enhanced [5]. According to Abdulahmeed, sex education is a major topic which must be educated to preschool teachers in the form of a course credit. Moreover, teachers can educate children on topics related to privacy and sexual abuse prevention programs [18].

From a medical point of view, masturbation has no negative effect on children. According to Birol et al., university studies have a more positive attitude toward child sexual behavior, indicating the importance of counseling courses provided in their final year of education. Results demonstrate that university students still hold social norms in their attitudes while passing their general courses. Nevertheless, they rethink their opinions upon passing counseling courses. As a result, educated teachers better understand children’s sexual desires and features [12].

The examination of the relationship between teachers’ knowledge and attitude on the one hand, and their demographic characteristics on the other revealed no correlation between them. However, a correlation existed between teachers’ attitudes on the one hand, and their level of education and marital status on the other. By increasing the level of education, teachers’ scores of attitudes regarding child sex education increase. Therefore, it is recommended that preschools employ teachers with a relevant educational background.

## Conclusions

Results of the present study showed that the implementation of the preschool sex education program for teachers promoted their knowledge and attitude. Nowadays, with changes in lifestyle, most children spend hours at preschools every day. Together with families, teachers play a key role in educating children. They require comprehensive education on topics related to child sex education. Participation in preschool sex education course familiarizes teachers with the stages of sexual development, formation of sexual identity, children’s sex-related questions at every age and how to respond to them, masturbation in children, the signs and characteristics of sexually abused children and how to behave with these children. Experts at the State Welfare Organization of Iran can use the findings of this study to design programs in the form of workshops, classes, and counseling programs on children sex education for preschool teachers.

## Data Availability

The datasets used and/or analysed during the current study are available from the corresponding author on reasonable request.

## Abbreviations

CVR: Content validity ratio
CVI: Content validity index
